# “KEEP AN EYE ON MY PLACE”: THE DYNAMICS OF SOCIAL CONNECTION FOR OLDER ADULTS LIVING IN SUBSIDIZED HOUSING

**DOI:** 10.1093/geroni/igae098.1961

**Published:** 2024-12-31

**Authors:** Thomas Cudjoe

**Affiliations:** Johns Hopkins University School of Medicine, Baltimore, Maryland, United States

## Abstract

Social isolation is a burdensome problem for low-income older adults. Many low-income older adults live in subsidized housing. However, few studies have examined this problem in this context. We sought to understand processes that may facilitate, or are barriers to social connections in older adults who live in this setting. We conducted in-depth semi-structured interviews with a purposeful sample of older adults living in subsidized housing communities in Baltimore City. Many residents had a small but meaningful residential network, facilitated by housing characteristics that support connection. Overall, though, residents were cordial but not connected. Challenges related to aging, the undesirable behaviors of others, and a desire for privacy limited social connections or hindered meaningful connection and led to social isolation within the housing communities.

